# Supplementary material for: Global adaptation to climate change in the twilight zone revealed by shared signals of selection in mesopelagic lanternfishes
Source: bioRxiv. 2026 May 26:2026.05.22.727234. Preprint. [Version 1] doi: 10.64898/2026.05.22.727234 (PMC13232121; doi:10.64898/2026.05.22.727234)
Supplement: Supplement 2 [file NIHPP2026.05.22.727234v1-supplement-2.pdf]

bioRxiv preprint doi: https://doi.org/10.64898/2026.05.22.727234; this version posted May 26, 2026. The copyright holder for this preprint (which was not certified by peer review) is the author/funder, who has granted bioRxiv a license to display the preprint in perpetuity. It is made available under a CC-BY 4.0 International license.

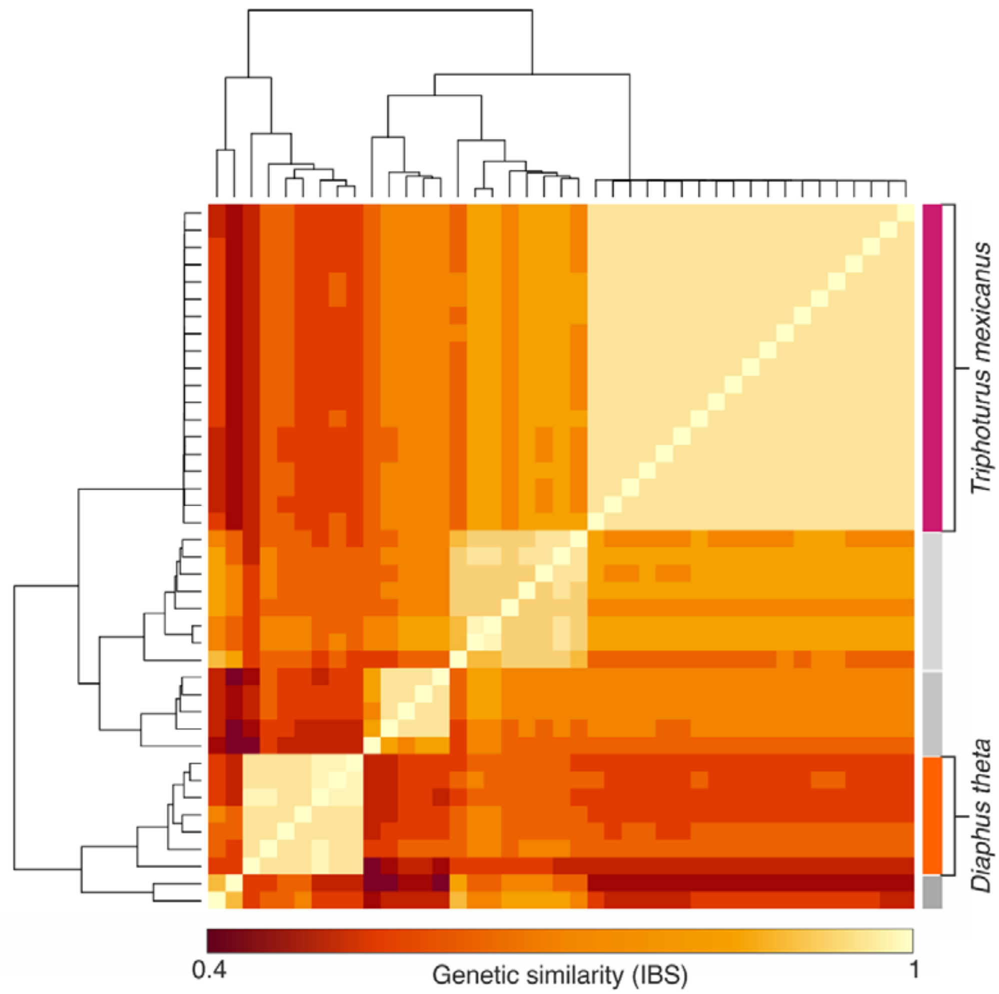

Supplementary Figure 1. Clustering of samples based on genomic similarity in the Lampanyctini and Diaphini clades across a set of independent SNPs, pruned for linkage disequilibrium using PLINK 1.9 (Purcell et al., 2007). This analysis was run without *a priori* awareness of species identity. Genetic similarity is given as identity-by-state (IBS), representing the proportion of shared alleles (no two samples shared less than 40% of alleles). Of the recovered clusters, two were used for downstream analyses based on robustness and number of specimens, corresponding to individuals later identified morphologically as *Triphoturus mexicanus* and *Diaphus theta*.

bioRxiv preprint doi: https://doi.org/10.64898/2026.05.22.727234; this version posted May 26, 2026. The copyright holder for this preprint (which was not certified by peer review) is the author/funder, who has granted bioRxiv a license to display the preprint in perpetuity. It is made available under a CC-BY 4.0 International license.

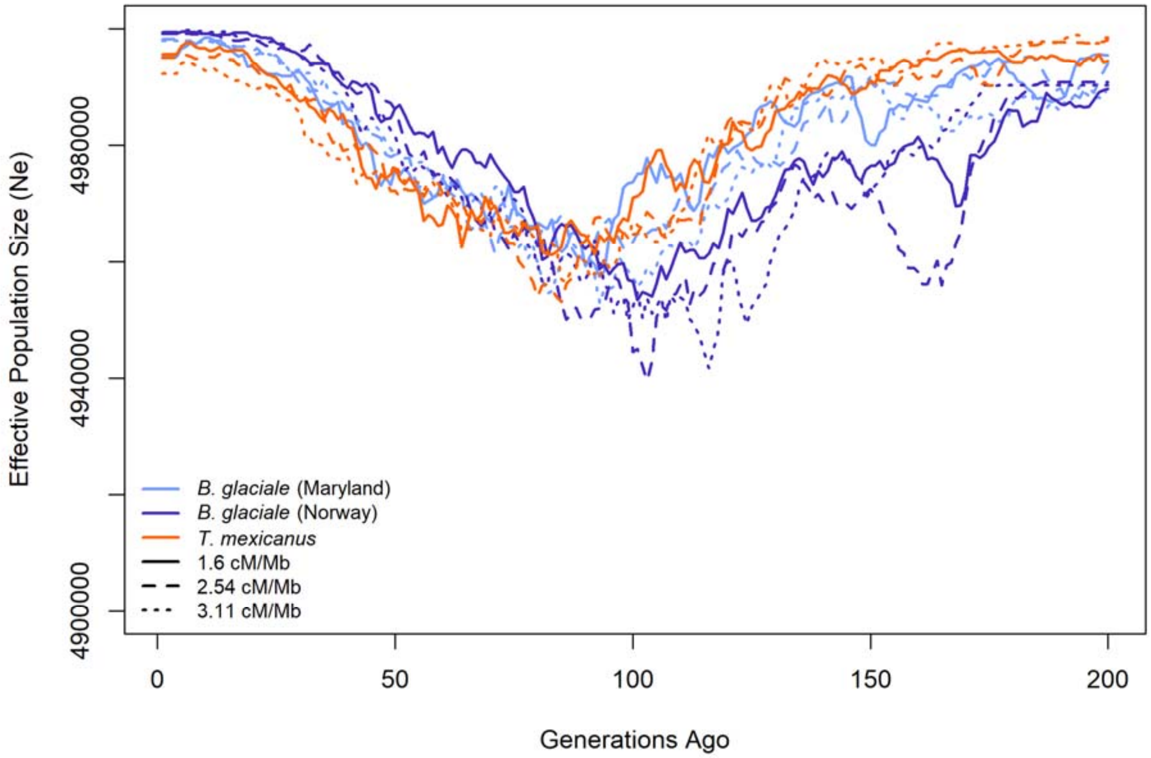

Supplementary Figure 2. Effective population size (N_e) as estimated by GONe (Santiago et al., 2025) over the past 200 generations in *Benthosema glaciale* from Norway and Maryland, and in *Triphoturus mexicanus*. As recombination maps are not available for myctophids, three different recombination rates were tested (assumed constant): 1.6 cM/Mb from the zebrafish (Bradley et al., 2011); 2.54 cM/Mb from the Atlantic herring (Pettersson et al., 2019); and 3.11 from the threespine stickleback (Wang et al., 2026). A minor fluctuation in N_e at -100 generations, consistent across all tests, is emphasized by the y-axis scale, but is likely not biologically meaningful given the magnitude of N_e (Gargiulo et al., 2024).
